# DIMENSIONAL DIAGNOSIS IN SCHIZOPHRENIA SPECTRUM DISORDERS: A CASE REPORT

**DOI:** 10.1192/j.eurpsy.2023.2219

**Published:** 2023-07-19

**Authors:** A. S. Cusenza, L. Zebi, G. Menculini, M. Grignani, F. Fontana, A. A. V. Tortorella

**Affiliations:** 1 Department of Psychiatry, University of Perugia; 2 Department of Mental Health; 3 Department of Mental Health, Local Health Unit “Umbria 1”; 4 Department of Mental Health, University of Perugia, Perugia, Italy

## Abstract

**Introduction:**

The use of diagnostic categories, although useful, fails in capturing the psychopathological complexity of the individual case. As for schizophrenia spectrum disorders, positive symptoms are not always included in the presentation, so further dimensions should be considered for a correct diagnosis.

**Objectives:**

To describe the importance of dimensional diagnosis in schizophrenia spectrum disorder based on a clinical experience

**Methods:**

We report the case of a late-onset schizophrenia spectrum disorder with an affective presentation

**Results:**

I. is a 44-year-old woman who accessed the Community Mental Health Center due to subjective memory complains. After clinical evaluation, depressive symptoms and circadian rhythm disturbances emerged. The patient also reported dissociative experiences, which emerged after her brother’s death. She underwent a neurological visit that excluded the possible early manifestation of a neurodegenerative disorder. Quetiapine was at first prescribed, due to the possible action on both insomnia and mood symptoms, with insufficient response. After a few visits, a deeper mental state examination revealed the presence of delusions. The patient also reported having experienced hallucinations. Psychotic symptoms appeared to be persistent and pervasive. We changed the antipsychotic to full-dose olanzapine, with good response. After a six-month observation, the patient was diagnosed with schizophrenia.

**Conclusions:**

The diagnosis of late-onset schizophrenia should take into account clinical history, drugs response, and the evaluation of different psychopathological dimensions

**Disclosure of Interest:**

None Declared

